# Supplementary material for: OsLRR-RLP2 Gene Regulates Immunity to Magnaporthe oryzae in Japonica Rice
Source: Int J Mol Sci. 2024 Feb 12;25(4):2216. doi: 10.3390/ijms25042216 (PMC10889788; doi:10.3390/ijms25042216)
Supplement: Supplementary file 1 [file ijms-25-02216-s001.zip › ijms-2841005-supplementary.pdf]

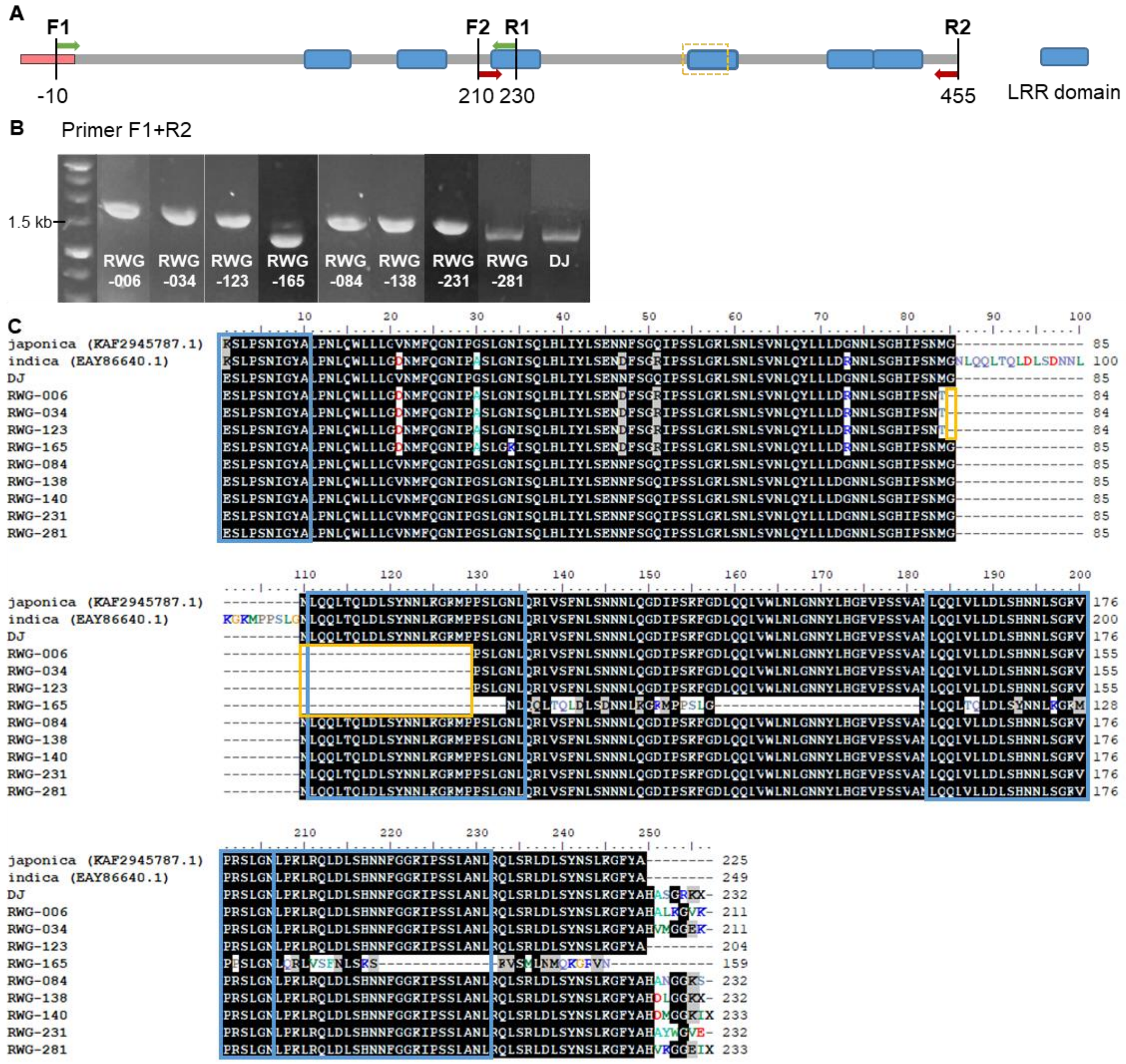

Figure S1. Amino acid sequence analysis of RWG lines at DNA level. (**A**) Position of primers used to assess the genotype of *OsLRR-RLP2*. Blue boxes indicate LRR domains. Yellow box indicates nonconserved amino acid residues in some RWG lines. Red box indicates upstream of *OsLRR-RLP2* gene. (**B**) PCR-based confirmation of DJ and 9 RWG lines using F1 and R2 primers. Lane 1: 1.5 kb DNA ladder. (**C**) OsLRR-RLP2 sequence confirmation of 9 RWG lines using F2 and R2 primers. Lines 4 to 7 are *indica* species, and lines 8 to 12 are *japonica* species. Blue boxes indicate LRR domain. Yellow boxes indicate nonconserved amino acid residues.

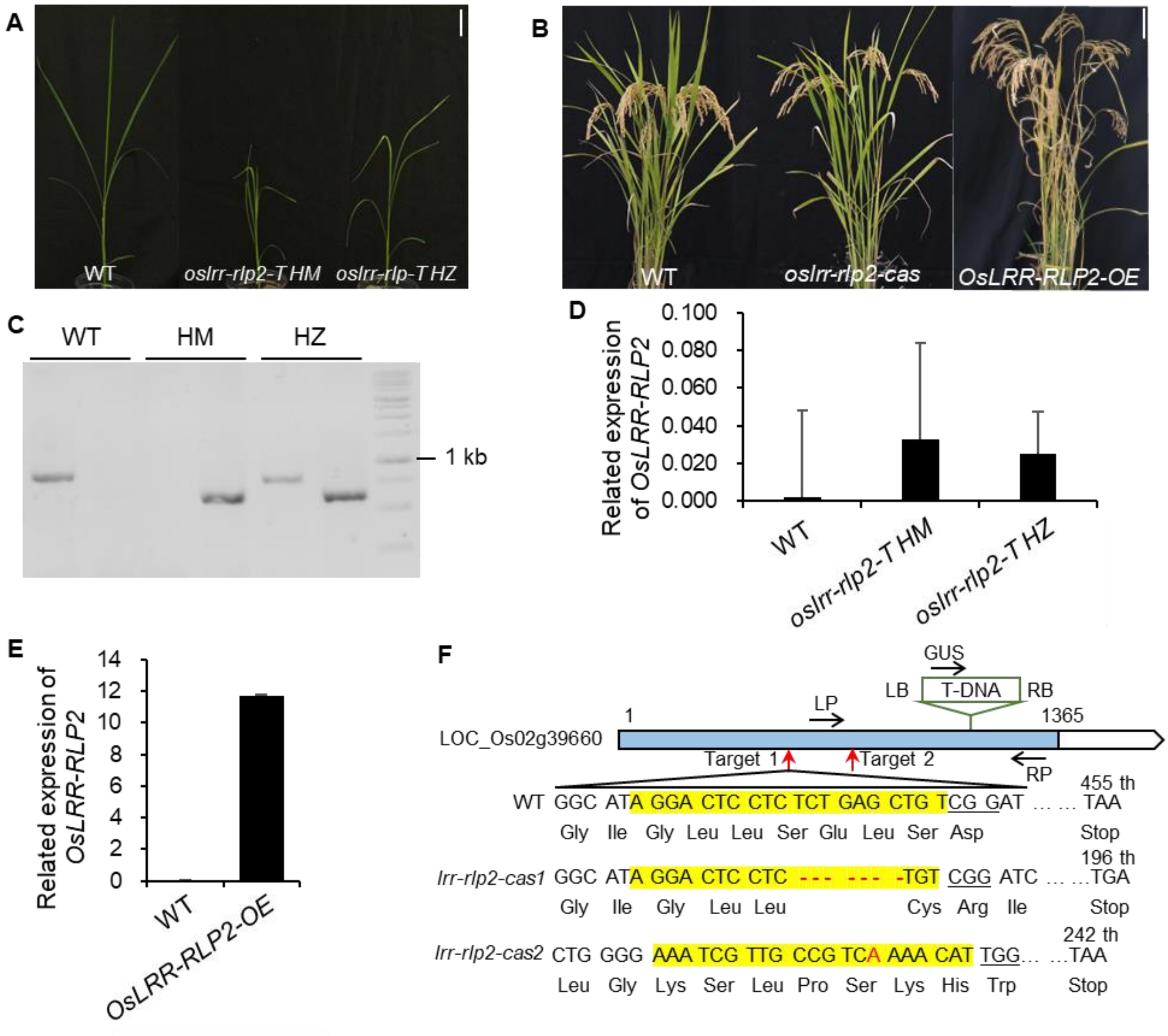

Figure S2. Generation of *OsLRR-RLP2* transgenic plants. (**A**) The phenotypes of WT, *oslrr-rlp2 T-DNA HM,* and *oslrr-rlp2 T-DNA HZ*. Scale bar = 5 cm. WT, wild-type; oslrr-rlp2-T HM, oslrr-rlp2 T-DNA homozygous; oslrr-rlp2-T HZ, oslrr-rlp2 T-DNA heterozygous. (**B**) The phenotypes of CRISPR/Cas9 and overexpressed plants at 4 months. Scale bar = 10 cm. WT, wild-type. (**C**) PCR genotyping analysis of T-DNA insertion mutant plants. Using genomic DNA as the template, PCR with internal primers was conducted for WT and T-DNA insertion mutants. Lane 7: 1 kb DNA ladder. (**D**) qRT-PCR analysis of *OsLRR-RLP2* gene expression level in T-DNA insertion mutant lines. Three technical replicates were performed, and error bars indicate ± SD. (**E**) qRT-PCR analysis of *OsLRR-RLP2* gene expression level in WT and *OsLRR-RLP2-OE*. The leaves were subjected to RNA extraction. Three technical replicates were performed, and error bars indicate ± SD. (**F**) Generation of homozygous mutant of *OsLRR-RLP2* through gene editing using CRISPR/Cas9 and T-DNA insertion (green box). Two target sites were disrupted in the first exon. Insertions and deletions (indels) are indicated in red arrow. T-DNA insertion homozygous plants were screened by PCR using LP, RP, and GUS primer. LP, Left primer; RP, Right primer.

Table S1. List of 9 accessions used for *M. oryzae* test, which contained OsLRR-RLP2 indels.

Cultivar name	Subspecies	Origin	Type	Line number	Indel number
CT9993-5-10-1-M	Indica	COL	Introduction	RWG-006	63
BALA	Indica	IND	Introduction	RWG-034	63
Chungdo Hwayang 14	Indica	KOR	Weedy	RWG-123	72
Milyang 23	Indica	KOR	Breeding line	RWG-165	72
Syalebyeo-163-1-B	Temperate japonica	KOR	Weedy	RWG-084	0
Jinbu Byeo	Temperate japonica	KOR	Breeding line	RWG-138	0
Hopyung	Temperate japonica	KOR	Breeding line	RWG-140	0
Gangchan	Temperate japonica	KOR	Breeding line	RWG-231	0
Jinbaek	Temperate japonica	KOR	Breeding line	RWG-281	0

Table S2. The primers used in this study.

Primer name	Forward primer sequense (5' to 3')	Reverse primer sequense (5' to 3')	Objective
OsLRR-RLP2_F1/R1	TGCTTTACCACTGTGCAGGT	TGGAGATGAATTTTCTGGGG	Genotyping
OsLRR-RLP2_F2/R2	GCGCCCTTTATAATCTCTCTTC	AACATGCCATTAGTGCGTGA	Genotyping
OsLRR-RLP2_T-DNA	TTATCTCTCGGGGCACATTC	CAAGCGACTGAGTTGACGAA	Genotyping
GUS	AACGCTGATCAATTCCACAG		Genotyping
OsLRR-RLP2_qRT	TCAGGACACATACCGTCCAA	CTTCAGCGGCTAGTGAGCTT	qRT-PCR
OsUbiquitin	GCACAAGCACAAGAAGGTGA	GCCTGCTGGTTGTAGACGTA	qRT-PCR
OsMoPot2	ACGACCCGTCTTTACTTATTTGG	AAGTAGCGTTGGTTTTGTTGGAT	qRT-PCR
